# Herpes simplex virus interference with immunity: Focus on dendritic cells

**DOI:** 10.1080/21505594.2021.1980990

**Published:** 2021-12-13

**Authors:** Farías MA, Duarte LF, Tognarelli EI, González PA

**Affiliations:** Millennium Institute on Immunology and Immunotherapy, Departamento de Genética Molecular y Microbiología, Facultad de Ciencias Biológicas, Pontificia Universidad Católica de Chile, Santiago, Chile

**Keywords:** Dendritic cells, HSV-1, HSV-2, immune evasion, immunity, T-cell, unfolded protein response (UPR), autophagy, apoptosis

## Abstract

Herpes simplex virus type 1 (HSV-1) and type 2 (HSV-2) are highly prevalent in the human population. These viruses cause lifelong infections by establishing latency in neurons and undergo sporadic reactivations that promote recurrent disease and new infections. The success of HSVs in persisting in infected individuals is likely due to their multiple molecular determinants involved in escaping the host antiviral and immune responses. Importantly, HSVs infect and negatively modulate the function of dendritic cells (DCs), key immune cells that are involved in establishing effective and balanced immunity against viruses. Here, we review and discuss several molecular and cellular processes modulated by HSVs in DCs, such as autophagy, apoptosis, and the unfolded protein response. Given the central role of DCs in establishing optimal antiviral immunity, particular emphasis should be given to the outcome of the interactions occurring between HSVs and DCs.

## Introduction

Herpes simplex viruses type 1 (HSV-1), and type 2 (HSV-2) are human viruses with linear double-stranded DNA genomes that belong to the *Alphaherpesvirinae* subfamily. Similar to other herpesviruses, HSVs elicit persistent infections. Importantly, HSVs can remain latent in neurons with sporadic or periodic reactivations depending on the individual. Infections caused by these viruses are widespread, with a global prevalence of approximately 67% for HSV-1 and 10% for HSV-2 [1,2]. Although most infected individuals are asymptomatic, up to 80–90%, they are nevertheless reservoirs and vectors for viral transmission onto new hosts, for example, through subclinical reactivations and unnoticed virus shedding [3]. HSVs can produce mild diseases such as ulcerative lesions in the orofacial and genital areas [3–5], and although orofacial lesions are usually associated with HSV-1 and genital ulcers with HSV-2, in recent decades genital herpes caused by HSV-1 infection has increased significantly in people aged 15–49 mainly in industrialized countries [6–8]. On the other hand, infections with HSVs can cause severe clinical manifestations such as permanent blindness, encephalitis, meningitis, or even death, despite treatment with antivirals that inhibit viral replication [9–11].

HSV particles consist of icosahedral capsids that encase the linear viral DNA genomes [3]. Capsids are surrounded by a coat of viral proteins called the tegument, which is further wrapped by a lipid bilayer that exposes numerous glycoproteins on the virion surface, which mediate virus entry into target cells [3]. The viral glycoprotein B (gB) of both viruses, as well as gC of HSV-1, bind to heparan sulfate receptors, which are present in most cells [12]. Glycoprotein G (gG), which is not directly related to virus entry into the cell also attaches to glycosaminoglycans on the cell surface [13,14]. Glycoprotein D (gD) in turn, which is needed for infection binds to nectin-1 or nectin-2 host molecules expressed mainly in nonimmune cells, or the herpesvirus entry mediator (HVEM), a tumor necrosis factor receptor (TNFR)-related receptor mostly expressed in immune cells [12,15]. This in turn activates the viral gH/gL protein complex and activates the fusiogenic properties of gB, which carries out the merging of the viral and host lipid membranes and allows the viral capsid and tegument proteins to enter the cell cytoplasm [16]. The capsids are then transported to the nucleus via microtubules, where the viral DNA is injected into this compartment through the docking of the capsids to nuclear pores [17]. Viral gene expression then occurs in sequential temporal waves: first immediate-early (IE or alpha) genes, then early (E or beta) genes, and later on, late (L or gamma) genes are transcribed [17]. The virus genomes encode not only structural determinants, but also numerous virulence factors aimed at counteracting the cellular antiviral response, as well as innate and adaptive immune components. Overall, this allows these viruses to successfully replicate in peripheral tissues and eventually expand on to neurons for lifelong persistent infection [17]. Indeed, viral particles produced during the lytic replication cycle in epithelial cells can reach the sensory and autonomic nerve terminals innervating the initial infection site and travel in a retrograde manner through axonal transport to the cell nucleus [18]. These neurons are mainly associated with sensory ganglia associated to the infection site, such as the trigeminal ganglia (TG) for HSV-1 and dorsal root ganglia (DRG) both, for HSV-1 and HSV-2 [19,20]. Once in the neuronal cell body, HSVs can enter a latent state from which they can reactivate under stress conditions, such as psychological stress, fever, and menstruation [19,21]. After an episode of productive reactivation, virions are transported in an anterograde manner along the microtubules to the primary infection site, causing recurrent lesions or asymptomatic virus spreading on to other tissues or new hosts [18,19,22].

Dendritic cells (DCs) are key immune components necessary for the initiation and regulation of immune responses against pathogens [23,24]. DCs act as immune sentinels by sensing antigens at the periphery of the organism or within internal organs and presenting their components to T cells as small peptides loaded on major histocompatibility complex molecules (MHC-I and -II) [25]. Notably, HSVs can infect DCs and interfere with numerous of their molecular and cellular processes, as discussed in detail below in this review, such as direct antiviral responses, apoptosis, autophagy, and the unfolded protein response (UPR).

Given the central role of DCs in initiating, polarizing, regulating, or dampening host antiviral responses, HSV interference with the phenotype and functions of these cells will likely have a significant impact on the host immune response against these viruses. Thus, focusing the attention on these DCs during HSV infection and understanding the outcome of DC-HSV interactions could have significant implications over the immune response to these viruses. Here we review and discuss the currently available literature regarding the impact of HSVs over DCs.

## Dendritic cell subtypes

**Figure 1.**
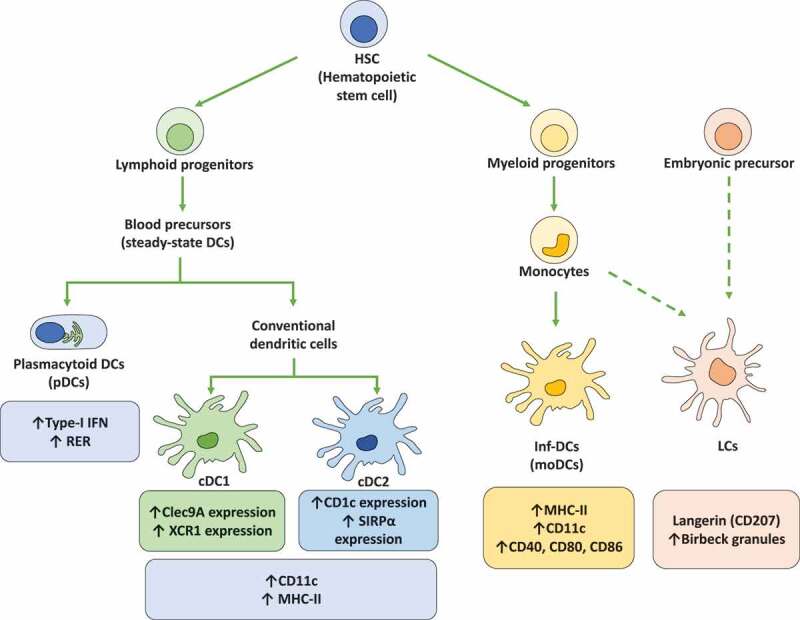
Dendritic cell subsets. Dendritic cells (DCs) are subdivided into different subsets that display different phenotypes and functions. Importantly, hematopoietic stem cells (HSCs) are the main precursors of the different DCs lineages. On the one hand, HSCs generate lymphoid progenitors that produce the differentiation of steady-state DCs that are subdivided into plasmacytoid DCs (pDCs) and conventional DCs (cDCs). Plasmacytoid DCs (pDCs) are characterized by the expression of high levels of type-I interferons (IFN-I) and the presence of high amounts of rough endoplasmic reticulum (RER). cDCs display a high expression of CD11c and MHC-II molecules. Moreover, cDCs are subclassified into cDC1 and cDC2, with cDC1 expressing high levels of the C-type lectin domain-containing 9A (Clec9A) and the chemokine XC receptor 1 (XCR1), while cDC2 express on their cell surface high levels of CD1c and the signal regulatory protein α (SIRPα). On the other hand, HSCs are the precursors of myeloid progenitors, which can be differentiated into monocytes and later into inflammatory DCs (inf-DCs), also called monocyte-derived DCs (MoDCs). Inf-DCs characterized by the expression of high levels of MHC-II, CD11c and costimulatory molecules (CD40, CD80, and CD86). Finally, langerhans cells (LCs) are specifically located in the epidermis and originate from monocyte precursors and embryonic precursors. LCs present langerin (CD207) expression and the expression of birbeck granules in the cytoplasm

Dendritic cells are subdivided into different subsets that differ in their phenotype and functions [26]. At present, three main groups of DCs have been described, namely steady-state DCs, inflammatory DCs (inf-DCs), and Langerhans cells (LCs) [26] (Figure 1).

The steady-state DCs, in turn, are subdivided into conventional DCs (cDCs) and plasmacytoid DCs (pDCs) [27]. cDCs are characterized by the expression of high levels of CD11c and MHC-II surface molecules [27]. Further, cDCs are divided into two subtypes, cDC1 and cDC2, where cDC1 are characterized by the expression of the C-type lectin domain-containing 9A (Clec9A) and the chemokine XC receptor 1 (XCR1), while cDC2 are characterized by high levels of expression of CD1c and the signal regulatory protein α (SIRPα) on the cell surface [26,28]. pDCs are mainly characterized by their ability to produce high amounts of type-I interferons (IFNs), as well as high levels of rough endoplasmic reticulum (RER) that provides these cells with a particular cell morphology [28].

Inf-DCs, also called monocyte-derived DCs (MoDCs), originate from monocyte precursors and mainly arise upon infection or injury followed by events of cytokine or chemokine production [28]. Inf-DCs display a high-level of MHC-II, CD11c, and costimulatory molecule expression, such as CD40, CD80, and CD86 [28]. LCs, in turn, are specifically located in the epidermis and are closely associated with keratinocytes [28]. LCs are also characterized by the expression of Birbeck granules and langerin (CD207) [26].

On the other hand, DCs can also be classified according to their migration capacities, such as resident or migratory DCs [28]. The resident DCs population is mainly found in the lymphoid organs during their lifespan [29]. In the steady-state, resident DCs have an immature phenotype characterized by a low expression of costimulatory molecules [29]. Resident DCs can be pDCs or cDCs subsets, such as CD8^+^ DCs and CD8^−^/CD11b^+^ DCs, respectively [30]. In contrast, migratory DCs mobilize from peripheral tissues and non-lymphoid organs into the corresponding draining lymph nodes, a process during which these cells acquire a mature phenotype; some migratory DCs are CD103^+^/langerin^+^/CD11b^+^ [29].

Finally, DC subsets can be classified according to their differentiation stage, such as immature (iDCs) or mature (mDCs) phenotypes. While iDCs express the CD11c marker but have low expression of MHC-II and costimulatory molecules, such as CD80, CD83, and CD86, mDCs show upregulated expression of these costimulatory molecules, as well as MHC-II [31].

## HSV infection of dendritic cell subsets

Dendritic cells have multiple functions that are related to regulating immune responses against pathogens [32]. Generally, DCs take up antigens using specialized surface receptors, such as endocytic receptors, phagocytosis receptors, and C-type lectin receptors, but also may do so through pinocytosis [28]. DCs express numerous extracellular and intracellular pattern recognition receptors that can sense a wide range of danger signals associated with pathogens or tumors, among others [33,34]. Once DCs interact with antigens, they can be activated and produce multiple cytokines and chemokines, as well as upregulate costimulatory molecules on their surface [28]. Subsequently, DCs process these antigens and can migrate to lymph nodes where they present protein-derived peptide fragments to CD4^+^ and CD8^+^ T cells [28].

Numerous studies have reported that both immature and mature MoDCs either from human or mouse origin are successfully infected with HSV-1 or HSV-2 *in vitro*, with iDCs and mDCs displaying viral immediate-early gene-related protein expression (e.g., ICP0, ICP4, ICP27), early gene (e.g., ICP8), and late gene (e.g., gB) expression with the corresponding proteins being produced [35–37]. iDC infection with HSVs results in a complete viral replication cycle with the release of infectious virions into the supernatants within 12 to 24 hours post-infection (hpi) [35]. On the other hand, mDC infection with HSVs also yields progeny virus, although seemingly at a lesser extent, and the viral particles are not abundantly released into the supernatant but mainly transferred to adjacent cells in a cell-to-cell contact-dependent manner [36]. The infection process in these DCs does not generate significant changes in the levels of the adhesion molecule CD38 or intercellular adhesion molecule-1 (ICAM-1), and the expression of the CD83 costimulatory molecule, as compared to HSV-1-infected iDCs [38]. In addition, during productive HSV infection, heavy particles (H) are generated, which are infectious virions with all virus components, such as viral DNA, capsid, tegument, and lipid envelope. Also, noninfectious light (L) particles are produced, which are viral particles that contain numerous HSV proteins but lack the capsid and viral DNA [39]. Regarding human mDCs, inoculation with HSV-1 mainly causes nonproductive infection associated with the release of L-particles [36]. The function of L-particles is not well understood to date, but at least they play a role in regulating the expression of the costimulatory molecule CD83 and the IL-6 receptor (IL6R) in DCs [40,41]. Mass spectrometry characterization of HSV-1 L-particles derived from human mDCs showed high levels of the ICP4, ICP6, gB, gD and UL42 proteins, and low levels of the ICP0 protein in these particles [42]. Furthermore, this study found that L-particles play a role in the regulation of DC function, by modifying surface CD83 expression, as well as impairing T cell activation [42]. Another study by Retamal-Díaz et al., found that HSV-2-infected DCs mainly release defective particles, apparently devoid of genetic material [43].

Regarding pDCs, an *in vitro* study suggests that this DC subset is resistant to HSV-1 and HSV-2 infection, as no viral transcripts and viral proteins were detected [44,45]. This study suggested that pDC resistance to HSV-2 was independent of the high levels of IFN-α that are usually produced by these cells [44]. Conversely, an *in vivo* study found that pDCs are susceptible to HSV-2 infection, and genital infection elicited pDC infiltration into the dermis at early and late stages of infection [44]. Although the *in vitro* studies indicate that pDCs are not susceptible to HSV infection, it has been observed that HSV-1 infection alters the expression of human pDC receptors involved in chemotaxis, antigen uptake, activation, maturation, migration, apoptosis, and costimulation [45]. Moreover, it was reported that HSV-1 upregulates CD8α expression in human pDCs, which characterizes a pDC subset that has only been reported in mice [46].

Additionally, LCs are susceptible to HSV infection; *in vivo* assays have reported that HSV-1 infection in the footpad produces an increase in the number of LCs, a process also observed in mucosal epidermis infected with this virus [47,48]. An increase in LCs at this site may indicate the activation of an immune response against HSVs, as the depletion of LCs led to enhanced HSV-1-related disease [47]. A similar result was also observed when CD11c^+^ DCs were depleted using CD11c-diphteria toxin (DT) in transgenic mice infected with HSV-1, which resulted in an increase in viral spread into the nervous system and an increased rate of morbidity and mortality [49]. Moreover, a recent study described that HSV-1 might infect a second epidermal DCs population, called epidermal cDC2s (Epi-cDC2s), closely related to dermal cDC2s [50]. *In vitro* studies have shown that Epi-cDC2s produce higher levels of HSV-1 replication than LCs, which is relevant considering that both DCs subtypes express similar levels of the HSVs entry receptors nectin-1 and HVEM and show similar levels of HSV-1 entry [50].

Collectively, HSVs can infect different subsets of DCs, both in *in vitro* and *in vivo* conditions, as well as during different stages of differentiation, either as iDCs or mDCs. Noteworthy, depending on the differentiation stage of the DCs in which infection with HSV occurs, different immune response outcomes could be generated. In the following sections, we review this process, including the modulation of costimulatory molecule expression by HSVs, the antiviral response elicited during infection, and cellular processes that are affected by HSVs, such as autophagy, UPR, apoptosis, and DC migration to lymph nodes. Altogether, these processes will finally lead to a particular DC imprint that will impact antigen presentation by DCs and modulate the activation of virus-specific T cells.

## Interference with DC function by HSVsHSVs downregulate DC costimulatory molecules

Costimulatory molecules are a set of membrane-bound molecules expressed on the surface of DCs that interact with receptors on the T cell surface and lead to their activation or inactivation [51]. HSV infection of DCs interferes with the expression of costimulatory molecules on the surface of DCs, which likely will have an impact on the capacity of these cells to mount an effective immune response in the host [52]. Indeed, immature human MoDCs infected with HSV-1 show downregulated CD1a, CD40, CD54 (ICAM-1), CD80, and CD86 molecules expression, which is not observed in these cells if exposed to UV-inactivated HSV-1, evidencing a virus replication-dependent effect [35]. Interestingly, the viral protein ICP22 of HSV-1 (Table 1) has been reported to directly mediate the downregulation of CD80 expression in murine bone marrow-derived dendritic cells (BMDCs) [53]. In addition, the γ34.5 protein of HSV-1 inhibits the phosphorylation of host transcription factors interferon regulatory factor 3 (IRF3) and p65/RelA through its TBK1 binding domain, which in turn mediates the downregulation of CD40, CD80 and CD86 expression in murine BMDCs [54].

**Table 1.** Herpes simplex virus proteins interfere with DC functionality

HSVs proteins	Cellular Process/Protein expression	DCs subsets (Human or murine)	References
ICP22 (HSV-1)	Reduces the expression of CD80 costimulatory molecule.	Murine BMDCs	[53].
γ34.5 (HSV-1)	Mediates the downregulation of CD40, CD80, CD86 costimulatory molecules through the inhibition of IRF3 and p65/RelA phosphorylation.	Murine BMDCs	[54].
	Blocks TLR-mediated DC maturation inhibiting MHC-II, CD86, IL-6, IL-12, IFN-α and IFN-β expression.	Murine iDCs	[69,70].
	Abolishes IFN-I production through direct binding to TBK-1, preventing IRF3 phosphorylation.	Murine iDCs	[70].
	Blocks NF-κB activation through the inhibition of IKKα/β phosphorylation.	Murine iDCs	[70].
	Antagonizes autophagosome maturation in a Beclin-Binding-Domain (BBD)-dependent manner.	Murine BMDCs	[131,136].
	Inhibits the activation of naïve T cells.	Murine iDCs	[69].
ICP0 (HSV-1)	Promotes the degradation of CD83 costimulatory molecule.	Human mature DCs	[56].
Deoxyuridine triphosphate nucleotidohydrolases (dUTPases) (HSV-2)	Produce the activation of NF-κB, promoting IL-6 and IL-8 secretion.	Human DCs.	[64].
Vhs (HSV-1).	Blocks NF-κB activation in the early phase of HSV-1 infection.	Murine BMDCs	[71,72].
	Decreases IL-6 receptor expression in infected and uninfected bystander cells.	Human mDCs.	[41].
gB, gD, gH/gL (HSV-1).	Promote IFN-α and IL-10 expression.	Human MoDCs.	[87].
ICP47 (HSV-1)	Inhibits MHC-I antigen presentation by the interaction with the transporter associated antigen presentation (TAP).	Human DCs from PBMCs	[181].

On the other hand, CD83, a costimulatory molecule that impacts the antigen-presentation capacity of DCs, through the regulation of endosomal processes, was also negatively affected by HSV-1 and HSV-2 infections [55]. In human mDCs infected with HSV-1, the degradation of cell surface CD83 expression was seen within 6 to 8 hours after infection and was mediated by proteasome-dependent degradation [56]. Moreover, human iDCs infected with HSV-2 have been reported to display a significant reduction in CD80, CD83, and MHC-II expression on the cell surface, and was shown to be dependent on viral gene expression; being the CD83 reduction modulated by proteasome-mediated degradation [37,57]. Further, the IE HSV-1 protein infected-cell protein 0 (ICP0) has also been reported to modulate CD83 degradation in human mDCs by a mechanism based on the activity of the E3 ubiquitin ligase of ICP0 [56,58]. Nevertheless, another study suggested that CD83 downregulation is independent of viral replication, as uninfected bystander mDCs also express reduced amounts of CD83 on their surface when cultured with infected HSV-1-infected DCs [40]. This latter effect was explained by the transmission of L-particles from HSV-1-infected mDCs to uninfected DCs [40].

In summary, HSVs modulate the expression of numerous costimulatory molecules on the DC surface, and distinct viral proteins have been identified as essential for these effects.

## HSVs modulate the host antiviral responses TLR responses to HSVs

Toll-like receptors (TLRs) are a group of pattern-recognition receptors (PRRs) that recognize pathogen-associated molecular patterns (PAMPs) [59]. TLRs are mainly expressed in immune cells and are found both, on the surface of the cells as homodimers or heterodimers (TLRs 1/2, 2/6, 2, 4, and 5), as well as within intracellular compartments (TLRs 3, 7, 8, and 9) [60]. Once TLRs are activated, their signaling cascades generally elicit the recruitment of the myeloid differentiation factor 88 (MyD88), TIR-domain-containing adapter-inducing interferon-β (TRIF), TIR-domain-containing adapter protein (TIRAP), or TRIF-related adapter molecule (TRAM) [61]. The recruitment of MyD88, TRIF, TIRAP, or TRAM generates signal transmissions from the IL-1 R-associated kinase (IRAK) adaptor that activates tumor necrosis factor 6 (TRAF6) [59]. After, TRAF6 may activate the mitogen-activated protein kinase (MAPK)-mediated activator protein-1 (AP-1) or the transforming growth factor β (TGF-β)-activated kinase 1 (TAK1), which stimulates IκB kinase (IKK)-mediated nuclear factor kappa B (NF-κB) and the interferon regulatory factors 3 and 7 (IRF3/7) [62]. Activation of these signaling cascades results in the promotion of the expression of proinflammatory cytokines and IFN molecules [59,62].

**Figure 2.**
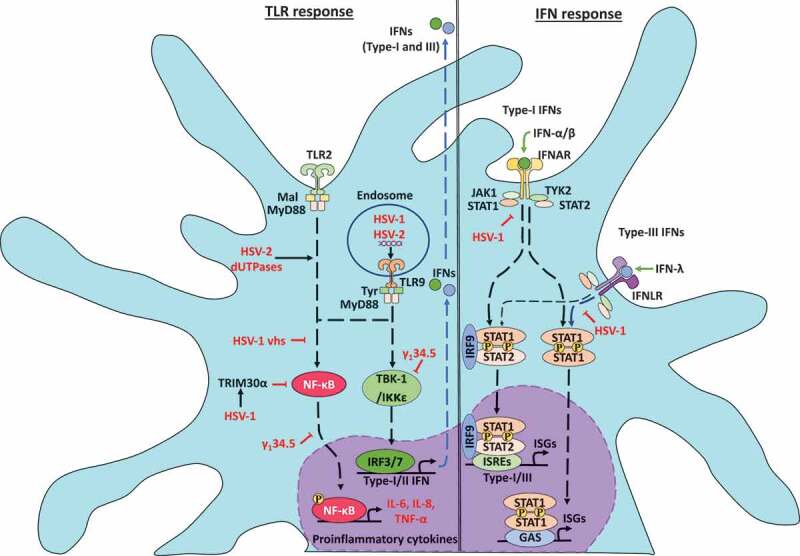
Herpes simplex viruses modulate early antiviral responses in dendritic cells. The toll-like receptor (TLR) responses to HSV infections in DCs is indicated on the left. HSV infection produces TLR2 and TLR9 activation. TLR2 signaling is modulated by HSV-2 deoxyuridine triphosphate nucleotidohydrolases (dUTPases) proteins promoting NF-κB activation and expression of proinflammatory cytokines IL-6 and IL-8. Moreover, in the endosome, TLR9 can sense HSV-1 and HSV-2 dsDNA and elicit myeloid differentiation factor-88 (MyD88) signaling cascades promoting the secretion of high levels of IFN-α, as well as the expression of TNF-α and IL-6. On the other hand, HSV-1 can inhibit TBK-1 activation through increased synthesis of the tripartite motif-containing-30α (TRIM30α), a protein that negatively regulates the activation of NF-κB signaling. Moreover, the HSV-1 vhs and γ_1_34.5 proteins block TLR-mediated NF-κB activation. Also, γ_1_34.5 can abolish IFN-I production through direct binding to TANK-binding kinase-1 (TBK-1), which prevents the interferon regulatory factor-3 (IRF3) phosphorylation and their nuclear translocation. On the right, interferon responses to HSV infections. HSV-1 can inhibit the signal transducer and activator of transcription-1 (STAT1) protein associated with the type-I IFN receptor (IFNAR) and type-III IFN receptor (IFNLR) that prevent the translocation of STAT1 complexes into the nucleus and the activation of the transcription of interferon-stimulated genes (ISG)

TLR2, TLR3, TLR4, and TLR9 activation are reported to be modulated by HSV infection in different cell types [61]. A study in HEK293T cells showed that the activation of a TLR2 response depended on HSV-1 laboratory strains, indicated by the authors as HSV-1TLR2* variants [63]. In the same study, the infection of murine cDCs with the HSV-1 TLR2* variants indicated that both, TLR2 and TLR9 sensors are activated with these variants and are associated with the secretion of proinflammatory cytokines IL-6 and IL-12 (Figure 2) [63]. Moreover, HSV-2 deoxyuridine triphosphate nucleotidohydrolases (dUTPases), which are metalloenzymes that catalyze the hydrolysis of dUTP to dUMP and pyrophosphate, produce the activation of NF-κB after the combination of TLR2/TLR1 heterodimers in HEK293 cells [64]. Further, in the same study, it was shown that human DCs treated with HSV-2 dUTPases generate TLR2 activation and produce an increase in the secretion of the inflammatory cytokines IL-6 and IL-8 [64,65].

On the other hand, HSV-2 DNA activates IFN-α secretion via TLR9 in murine pDCs [66]. Mechanistically, TLR9 can sense HSV-2 genomic dsDNA in infected-pDCs and elicits a signaling cascade regulated through the MyD88 adaptor molecule that concludes in the secretion of high levels of IFN-α [66]. Consistently, HSV-1 induces IFN-α synthesis in infected-pDCs via TLR9 dependent-activation. Experiments with pDCs from TLR9^−/-^ mice have shown significantly less IFN-α and IL-12 production than with pDCs from WT mice [67]. Importantly, IFN-α levels were reduced in TLR9^−/-^ pDCs infected with HSV-1, but not in MyD88^−/-^ pDCs, suggesting that TLR9 expression, but not MyD88 is required for the activation of pDCs by HSV-1 infection [68]. Nevertheless, in contrast with these results, TLR9-deficient cDCs produced similar levels of IFN-α in response to HSV-1 infection but reduced levels of tumor necrosis factor-alpha (TNF-α) and IL-6, compared with WT cDCs [68]. These results indicate that TNF-α and IL-6 production are partially dependent on TLR9 activation, but IFN-α production is independent of TLR9 [68].

On the other hand, HSV proteins such as γ_1_34.5 block TLR-mediated DC maturation by producing the inhibition of MHC-II, CD86, IL-6, IL-12, IFN-α, and IFN-β expression in iDCs (Table 1) [69,70]. Moreover, the HSV-1 vhs protein blocks NF-κB activation, but not IRF3 activation in the early phase of infection of murine BMDCs [71,72]. Further, studies with BMDCs infected with mutant HSV-1 lacking the vhs protein showed elevated secretion of IFN-α/β compared to BMDCs infected with WT HSV-1, suggesting that the vhs protein also blocks the release of proinflammatory cytokines in BMDCs infected with HSV-1 [71].

## HSV infection regulates interferon signaling pathways in DCs

Interferons are a group of cytokines organized in three different families that modulate the immune response and significantly affect pathogen infection and control [73]. The type-I IFN family comprises the extensively studied IFN-α and IFN-β cytokines, as well IFN-ɛ, IFN-τ, IFN-κ, IFN-ω, IFN-δ and IFN-ζ, some of these being somewhat poorly known [73]. Meanwhile, the type-II IFN family comprises only IFN-γ. On the other hand, the type-III IFN family consists of IFN-λ1, IFN-λ2, IFN-λ3 (or interleukin-29 (IL-29), IL-28A, and IL-28B, respectively) and IFN-λ4 [74].

The induction of IFN responses start with the recognition of pathogen products by a wide range of cell-surface and intracellular pattern recognition-receptor (PRRs), such as TLRs indicated above, retinoic cis-inducible gene I (RIG-I)-like receptors (RLRs), and NOD-like receptors (NLRs), among others [73,75]. After the binding of type-I IFNs to the IFNAR receptors (IFNAR1 and IFNAR2), IFN-γ to IFNGR, and type-III IFNs to the IFNLR heterodimer composed by IFNLR1 (also known as IL28Rα) and the subunit-β of the IL-10 receptor (IL-10Rβ), different downstream signaling pathways can be induced [76].

Canonical type I IFN signaling through IFNARs activates the signal transducers Janus kinase 1 (JAK1) and tyrosine kinase 2 (TYK2) [76], which in turn activate the signal transducer and activator of transcription molecules 1 and 2 (STAT1/STAT2), leading to the formation of the STAT1-STAT2-IRF9 (IFN-regulatory factor 9) complex. Then, the STAT1-STAT2-IRF9 complex is translocated to the nucleus to initiate the transcription of interferon-stimulated genes (ISGs), by binding to IFN-stimulated response elements (ISREs) [77,78]. On the other hand, the non-canonical type I IFN pathway induces the formation of STAT1 homodimers that translocate to the nucleus to bind to ISREs, or gamma-activated sequences (GASs) to promote the transcription of ISGs [79]. Moreover, IFN-I (IFN-α and IFN-β) can signal via cytokine-mediated signaling pathways, such as those mediated by STAT3, STAT4, STAT5 and STAT6, with the phosphorylation and dimerization of these factors being shown to induce the activation of repressor activator protein 1 (Rap1), Crk-like protein (CrkL), multiple mitogen-activated protein kinases (MAPK), insulin receptor substrate 1 (IRS-1) and 2 (IRS-2), Ras-related C3 botulinum toxin substrate 1 (RAC1) and phosphoinositide 3-kinase (PI3K) transduction pathways [76,80].

Regarding the classical signaling pathway by type-II IFNs, the interaction between IFNGR (IFNGR1 and IFNGR2) with IFN-γ induces the activation of JAK1 and JAK2, resulting in the formation and phosphorylation of STAT1 homodimers that translocate to the nucleus, where they bind to ISREs or GASs, that leads to the transcription of ISGs [79,80]. In addition, alternative type-II IFN activation pathways have been described, including via STAT4, the extracellular signal-regulated protein kinases 1 and 2 (Erk1/2), protein-tyrosine kinase 2-beta (Pyk2) and CrkL [80].

Finally, type-III IFNs activate IFNLR-IL10Rβ, which is associated with JAK1 and TYK2 signal transducers, and IFNGR signaling associated with JAK1 and JAK2 signal transducers [76]. Similar to type-I IFNs, type-III IFN receptor signaling promotes JAK-STAT signaling and the transcription of ISGs, such as MX dynamin-like GTPase 1 (MX1), interferon-induced protein with tetratricopeptide repeats 1 (IFIT1) and interferon-stimulated gene 15 (ISG15) [77,78].

## HSV modulation of interferon synthesis

The expression of interferons in DCs infected with HSVs is mainly regulated by type-I (IFN α/β) and type-III (IFN-λ) IFNs (Figure 2) [81,82]. An *in vitro* study using D2SC/1 DCs, which is a murine cell line, reported the production of IFN-α and IFN-β by these cells after infection with HSV-1, and a similar result was described for HSV-1-infected pDCs [83,84]. Another study reported that human MoDC infection with HSV-1 strain 17syn^+^, but not strain KOS, induced increased levels of cytokine expression for IFN-α/β, IL-28, IL-29, TNF-α, and chemokines CCL5 and CXCL10 at 9 hpi, yet at 18 hpi no increase was observed [85]. Notably, this process depended on viral replication as the inoculation of DCs with UV-inactivated HSV-1 did not elicit this effect [85]. Moreover, it is believed that IFN-I secretion by human-MoDCs-infected with HSV-1 might be mediated by the bystander activation of uninfected DCs, through IL-12p40 and IL-12p70 [86]. However, MoDC recognition of viral glycoproteins gB, gD, gH/gL produces an up-regulation of IFN-α and IL-10, but not IL-12p70 [87]. On the other hand, a study found that the mRNA expression of IFN-β, IL-6, and CXCL10 decreased in DCs infected with HSV-1-d109, an HSV-1 mutant virus that has deleted ICP0, ICP4, ICP22, ICP27, and the ICP47 IE genes [88,89]. Thus, IFN secretion associated with HSV infection seems to depend on IE gene expression.

Regarding HSV-2 infection, expression of IFN-α/β and IFN-λ was reported in infected-murine BMDCs, an effect that was also observed in HSV-2-infected murine splenic cDCs and pDCs [90]. Interestingly, the IFN-I response observed in cDCs infected either with HSV-1 or HSV-2 occurred independently of viral replication. Nevertheless, it depended on viral entry because using a virus lacking gL (ΔgL) did not produce an IFN-I response [90]. Moreover, the use of an UL15 mutant, which fails to package viral DNA into its capsid, displayed a reduced IFN-I response indicating that recognition of viral DNA or certain steps related to viral genome processing is needed for inducing effective IFN-I response [91]. On the other hand, MoDCs infection by HSV-2 produced a significant increase in TNF-α and IL-6 expression, with a reduced expression of IFN-β and IL-12p70, and a modest synthesis of IL-10 [57]. Furthermore, an *in vivo* study using the transgenic CD11c-DTR-tg mouse strain showed that the depletion of CD11c^+^ cells reduces IFN-I and IFN-III levels in vaginal wash samples revealing that DCs are the main source of IFN-I and IFN-III during genital infection with HSV-2 [92].

Regarding pDCs, these cells have been identified as potent secretors of IFN-I in response to numerous viruses. In line with this observation, *in vitro* studies have reported that pDCs secrete high levels of IFN-I after inoculation with infectious or UV-inactivated HSV-1 and HSV-2, through a TLR9 response, and display PLSCR1 and RNase 7 expression [66,93–95]. *In vivo* experiments have also shown early IFN-I production in murine pDCs, but not cDCs obtained from HSV-1- and HSV-2-infected mice, which is a process that depends on the activation of TLR9 [91]. Meanwhile, the subsequent IFN-α/β response was derived from numerous cell types, namely pDCs, cDCs, macrophages and fibroblasts, and was induced independently of TLR9 activation [91]. Seemingly, IFN-I upregulation in pDCs infected with HSV-1 would be mediated by increased IRF7 expression [96,97]. On the one hand, HSV-1 upregulates IRF-7 expression in human pDCs by an NF-κB-activation mechanism [96], but also HSV-1 increases IRF7 expression by the zinc finger CXXC family epigenetic regulator CXXC5 [97]. Interestingly, their genetic ablation produced the methylation of the IRF7 gene, which subsequently impaired IRF7 expression and ultimately elicited a decrease in the overall IFN-I response, allowing effective HSV-1 replication [97]. In contrast, an *in vivo* study reported that while pDCs were not essential for the early expression of IFN-I and proinflammatory cytokines after a vaginal infection with HSV-2 or subcutaneous HSV-1 infection, these cells were nevertheless relevant for mounting an effective immune response against HSV-1 and HSV-2 during systemic infections [98].

Another host factor that modulates IFN responses in the context of DC infection by HSVs is ankyrin repeat domain 1 (ANKRD1) [99]. ANKRD1 is a protein induced by inflammatory cytokines and can interact with NF-κB, IRF3, and IRF7 [99]. This protein is upregulated in DCs infected with HSV-1 and significantly increases HSV-1 viral loads when silenced in DCs, reportedly through a decrease in IFN-I and IL-29 production, suggesting that ANKRD1 is involved in innate antiviral immune signaling pathways in these cells [99].

Finally, IFN-I signaling has been reported to be critical for controlling HSV replication in DCs, as IRF-3^−/-^ murine BMDCs were shown to display increased HSV-1 replication that was associated with delayed IFN-I synthesis, and pretreating these cells with IFN-I resulted in reduced viral titers in the supernatants of these cells [72]. Moreover, BMDCs lacking both IFN-α and IFN-β signaling capacity produced more virus, at a similar level than IRF3^−/-^ BMDCs, suggesting that type-I IFN signaling is responsible for controlling HSV-1 replication in these cells [72].

## HSV downregulation of the interferon response

Regarding IFN downregulation by HSVs, recent studies have shown that many tripartite motif-containing (TRIM) proteins, a kind of E3 ubiquitin ligases, serve as critical regulators of the innate immune response during the infection of DCs with HSV thanks to the modulation of cytokine signaling processes related to IFN-I and TNF-α, or PRRs, such as TLR and RIG-I receptors [100–102]. It was recently identified that HSV-1 and dsDNA derived from HSV-1 suppress innate immune responses in mDCs and BMDCs, through TRIM29, leading to the downregulation of IFN-α, IFN-β, IL-6, and TNF-α expression [102]. On the other hand, the expression of host TRIM30α, a protein that negatively regulates NF-κB activation by targeting the TAK1 complex for degradation, is increased upon HSV-1 infection in BMDCs producing a decrease in IFN-α/β and IL-6 secretion, which was mediated through the stimulator of interferon genes (STING) signaling pathway that depended on its RING domain [101,103].

On the other hand, a recent study found that human mDCs infected by HSV-1 negatively modulate the expression of IL6R, a process that was also observed in uninfected bystander mDCs [41]. Mechanistically, IL6R downregulation in uninfected bystander DCs was associated with the transfer of HSV-1-derived noninfectious L-particles produced during infection of mDCs. The use of a neutralizing antibody, which blocked the transfer of L-particles to bystander mDCs, produced the recovery of the surface expression of IL6R on bystander mDCs [41]. In addition, this study suggested that the vhs protein plays a role in ILR6 expression in bystander mDCs, as the infection of mDCs with an HSV-1 virus that lacks this viral protein showed partial recovery of the expression levels of IL6R in bystander DCs [41].

Regarding other viral proteins, it has also been reported that the γ_1_34.5 protein hampers NF-κB activation through the inhibition of p65/Rel phosphorylation (Table 1) [70]. Accordingly, studies with a mutant virus for γ_1_34.5 showed activation of IκB kinase and p65/ReLA phosphorylation, resulting in NF-κB nuclear translocation. Noteworthy, NF-κB translocation promotes the activation of CD8^+^ DCs, but not CD8^−^ DCs, an effect mirrored by the high secretion of IL-6 and IL-12 levels by CD8^+^ DCs [104].

Importantly, HSV-1 has also been reported to downregulate IFNGR1 expression on the surface of virus-infected human mDCs (Figure 2) [105]. Furthermore, HSV-1 infection was shown to inhibit the IFN-γ-induced tyrosine phosphorylation of STAT1, but not interfere with the STAT1 expression. As a direct downstream effect over STAT1 phosphorylation, the activation of IRF-1 was also strongly reduced in mDCs infected with HSV-1, and these processes were shown to be dependent on the replication of HSV-1, as once inactivated by UV inoculation with the virus did not downregulate IFNGR1 expression, STAT1 phosphorylation or IRF-1 expression [105]. Other studies using BMDCs obtained from STAT1-deficient mice found that these DCs were more susceptible to HSV-1 infection than BMDCs obtained from WT mice, as they produced higher amounts of virus suggesting that the JAK-STAT1 pathway is involved in blocking HSV-1 replication in DCs [72,106].

Overall, HSVs modulate through several mechanisms IFN signaling pathways, either by interfering with the activation of signaling receptors or through the interaction with transcription factors that activate ISGs.

## HSVs induce dendritic cell apoptosis

Apoptosis, or programmed cell death, is a cellular process vital for tissue homeostasis, which is also essential for the development and function of an adequate immune system [107]. Dysregulation of apoptosis can have severe consequences for the individual, such as neurodegenerative diseases, autoimmune disorders, and inadequate immunological responses against pathogens [107–109].

Both extrinsic and intrinsic signaling pathways induce apoptosis, with the activation of a family of aspartate-specific cysteinyl proteases known as caspases [110]. The extrinsic pathway is associated with cellular responses to external stimuli that induce the activation of pro-caspase 8 to caspase-8 after ligand binding to membrane death receptors, such as the TNFR family (FAS (also called CD95) by TNFR1 proteins, such as TNF-α and TNF-related apoptosis-inducing ligand (TRAIL) [111]. After the activation of one or more of these receptors, their intracellular domains recruit the adaptor protein Fas-associated via death domain (FADD), or TNFRSF1A via the death domain (TRADD) that promotes the recruitment of pro-caspase 8 in the death-inducing signal complex (DISC) [111]. Then, pro-caspase 8 monomers accumulate at DISC, eliciting their dimerization and activation, which leads to the activation of caspase-8 and later, caspase-3 that is then able to induce apoptosis [111].

On the other hand, the activation of the intrinsic pathway depends on intracellular stimuli, in which case an increase in the activity of pro-apoptotic BH3-only proteins, such as the tumor suppressor p53 protein that bind to a pro-survival Bcl-2 protein family, control the activation of effectors of apoptosis, namely Bcl-2-associated X protein (BAX) and the Bcl-2-antagonist killer (BAK) [112,113]. Once BAX and BAK are activated, they induce the permeabilization of the mitochondrial outer membrane, promoting the release of cytochrome c (Cyt c) and the activation of caspase-9, which finally entails the activation of caspase-3 for inducing apoptosis [114].

**Figure 3.**
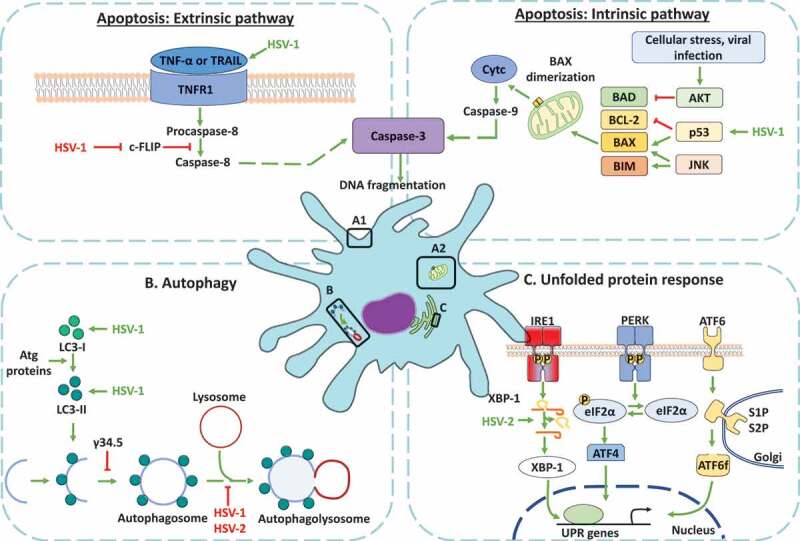
Dendritic cell cellular responses in response to infections with herpes simplex viruses. A. Apoptosis activation in dendritic cells infected by HSVs. A.1 Modulation of the extrinsic apoptosis pathway in DCs infected by HSVs. HSV-1 promotes the expression of death ligands, such as TNF-α and the tumor necrosis factor-related apoptosis-inducing ligand (TRAIL) recognized by the tumor necrosis factor receptor (TNFR), promoting a signaling cascade that concludes in the activation of caspase-8 and caspase-3 proteins. The activation of caspase-8 is negatively modulated by the cellular FLICE-inhibitory protein (c-FLIP). However, HSV-1 infection produces the inhibition of c-FLIP and the subsequent activation of caspase-8, promoting caspase-3 activation and the DNA fragmentation in DCs. A2. Modulation of apoptosis through the intrinsic pathway in DCs infected with HSVs. HSV-1 infection generates the up-regulation of p53, a protein that activates Bcl-2-associated X protein (BAX). Once BAX is activated, its dimerization in the mitochondrial outer membrane promotes mitochondrial membrane permeabilization and the release of cytochrome c (Cytc). Cyt c plays an essential role in the activation of caspase-9, allowing caspase-3 activation for inducing DNA fragmentation. B. Regulation of autophagy in DCs infected with HSVs. HSV-1 induces an increase in the expression of proteins associated with autophagosome formation, such as microtubule-associated protein-1 light chain 3 (LC3-I) and LC3-II formation. However, autophagosome formation is inhibited by the γ_1_34.5 viral protein. Moreover, the maturation of autophagosomes which consists of their fusion with lysosomes is inhibited by HSV-1 and HSV-2 infection in DCs. C. Modulation of the unfolded protein response (UPR) in DCs infected with HSV. The UPR response is regulated by three sensors called inositol-requiring protein 1α (IRE-1α), protein kinase RNA (PKR)-like ER kinase (PERK), and the activating transcription factor 6 (ATF6). IRE-1α is located in the endoplasmic reticulum (ER) and catalyzes the non-canonical splicing of X-box binding protein 1 (XBP-1) mRNA. HSV-2 infection modulates XBP-1, producing an increase in its splicing in DCs. Conversely, HSV-2 infection in DCs does not modulate the PERK and ATF6 sensors

DC infection with HSV-1 or HSV-2 induces apoptosis through caspase-3 activation in murine DCs (Figure 3(a)) [115]. Assays with terminal deoxynucleotidyl transferase-mediated dCTP biotin nick end labeling (TUNEL assay) showed a decrease in the cellular metabolic activity and DNA degradation in these cells during late stages of infection [115,116]. Additionally, a study using LCs from cultured skin reported a significant increase in apoptosis in HSV-2-infected langerin^+^/CD11c^+^/DCs, as compared to bystander LCs from HSV-1-infected mice or LCs from mock-infected mice [117]. HSV-1 and HSV-2 infection of human MoDCs was also associated with high induction of caspase-3 activation [118].

Regarding the extrinsic pathway of apoptosis (Figure 3(a1)), human iDCs infected with HSV-1 displayed increased expression of death ligands, such as TNF-α and TRAIL, but not Fas ligand (CD95L), as may have been expected [119]. Further, apoptosis analyses of human iDCs infected with HSV-1 show a downregulation of the cellular FLICE-inhibitory protein (c-FLIP), a potent inhibitor of caspase-8-mediated apoptosis, which produced a modest decrease in caspase-3 and a slight increase in caspase-8 expression [57,119,120]. Particularly, in DCs infected with HSV-1, c-FLIP deregulation occurs in a proteasome-independent manner, and the decrease in c-FLIP that has been observed does not depend on a reduction in gene transcription or mRNA stability, as c-FLIP mRNA increased upon infection [119,120]. Therefore, the authors have suggested that a possible mechanism of apoptosis by HSV in these cells may be through the downregulation of c-FLIP function [120].

Regarding intrinsic apoptosis pathways (Figure 3(a2)), analyses of human iDCs infected with HSV-1 evidenced an up-regulation of p53 that could be related to the activation of BAX, which promotes mitochondrial membrane permeabilization and finally results in caspase-3 activation and, thus, DC apoptosis [57,119,120].

Notably, it is understood that increased apoptosis in DCs will limit antigen availability for T cells which will favor HSV escape from the immune system [114]. Interestingly, apoptosis in DCs is in sharp contrast with what is observed during HSV infection in epithelial or neuronal cells, in which these viruses inhibit apoptosis, likely to promote high yields of infectious viral particles [121,122]. Further, the deletion of receptors involved in the phagocytosis of apoptotic cells (also called efferocytosis), increases DC apoptosis by HSV-1 infection and is associated with defective viral antigen-specific CD8^+^ T cell activation [123]. On the other hand, bystander DC phagocytose apoptotic HSV-2-infected DCs, a process that allows uninfected DCs to process viral antigens that enable the stimulation of HSV-specific CD8^+^ T cells [118]. Therefore, DC infection by HSVs regulates both components of extrinsic and intrinsic apoptosis pathways and impairs the presentation of viral antigens to T cells and consequently their optimal activation.

## HSVs modulate autophagy-related processes in DCs

Autophagy is a cellular process that participates in the clearance of aggregated or misfolded proteins, the adaptation of the cell to nutritional starvation, as well as the regulation of growth, aging, and cellular differentiation. However, it also acts as a defense mechanism against several pathogens [124,125]. Autophagy is a process with several defined steps that lead to the formation of autophagosomes and the fusion of these organelles with lysosomal membranes to form autolysosomes [126]. In DCs, an autophagy process called macroautophagy has been described in more detail than microautophagy and chaperone-mediated autophagy [127]. Macroautophagy is involved in the regulation of several DCs functions, such as DC maturation, TLR stimulation, antigen presentation, cytokine production, DC migration, and T cell activation [127]. Importantly, autophagy has been reported to be a process modulated by HSVs during the infection of epithelial and neuronal cells, and DCs as a mechanism to counteract the host antiviral response [19,128,129].

The molecular mechanisms inducing autophagy are cell type dependent in HSV infection [129,130]. Regarding DCs (Figure 3(b)), a study that used BMDCs reported that HSV-1 induces an increase in autophagy markers, such as microtubule-associated protein 1 light chain 3 (LC3) foci and LC3II in a protein kinase R (PKR)/eukaryotic translation initiation factor 2-alpha (eIF2α)-independent manner [129]. This study found that HSV-1-induced autophagy was independent of viral replication because inoculation with UV-inactivated HSV-1 also induced an increase in the levels of LC3II [129]. However, this induction depended on HSV-1 entry into the cells because BMDCs inoculated with entry-incompetent gH- and gB-HSV-1 mutant viruses did not display increased autophagy markers [129]. Moreover, the presence of cytosolic HSV-1 DNA, as well as the transfection of dsDNA oligonucleotides derived from the HSV-1 genome (60 mer), were reported to trigger LC3II formation, a process which was mediated in a STING-dependent manner [129]. These latter results add to a study with HSV-1 L-particles and a UL15 mutant that has capsids that lack the HSV-1 genome, and that were unable to stimulate LC3II formation as well [129].

Another study using the BMDC-derived immortalized cell line DC2.4 also reported an increase in LC3-II levels after infection with HSV-1 [131]. However, this LC3-II accumulation was not associated with the maturation of autophagosomes [131]. Interestingly, DC2.4 cell infection with HSV-1 interfered with the catabolic breakdown of p62, a protein involved in the autophagic flux, suggesting that HSV-1 interferes with the maturation of the autophagic process through the intracellular accumulation of p62 [131,132].

On the other hand, a study with iDCs and mDCs reported that HSV-1 induces autophagy in both DCs subsets, yet autophagy flux only occurred in iDCs, which was inhibited by the ablation of the kinesin family members KIF1B and KIF2A, which affected the autophagosome-lysosome fusion step [133,134]. Furthermore, it was shown that autophagy promoted lamin A/C, B1, and B2 degradation in HSV-1-infected iDCs, a phenomenon that did not happen in mDCs [135]. Notably, this process was found to facilitate the nuclear egress of progeny viral capsids into the cytoplasm and, consequently, the formation of new infectious particles [135].

At present, the viral protein γ_1_34.5 (Table 1) has been identified as the central molecular modulator of autophagy inhibition in epithelial and neuronal cells, namely through the dephosphorylation of eIF2α and the inhibition of beclin-1 protein, that controls autophagosome formation and autophagic flux [129]. However, a study demonstrated that HSV-1 induces autophagy in BMDCs through a mechanism dependent on STING, but not by the phosphorylation of eIF2α, nor their regulation by the γ_1_34.5 viral protein, because a γ_1_34.5 HSV-1 mutant virus also induced LC3-II accumulation in these cells, just like with the WT virus [129]. Other studies have found that γ_1_34.5 did not inhibit the induction of autophagy in DCs. However, this protein likely antagonizes the maturation of autophagosomes, based on the observation of p62 intracellular accumulation [131,136]. Further, the inhibition of autophagosome maturation would depend on the binding of γ_1_34.5 with beclin-1, because experiments with a virus lacking the Beclin-binding domain (BBD) in γ_1_34.5 decreased the levels of p62 [131].

Importantly, the role of the γ_1_34.5 viral protein in the inhibition of autophagosome maturation in DCs likely translates into alterations in the stimulation and activation of CD4^+^ and CD8^+^ T cells because disrupting the autophagosome in DCs will impact the presentation of antigens to T cells [131,136]. Moreover, studies validating the role of this viral protein in the modulation of autophagy in antigen processing and presentation have been obtained with DCs infected with a γ34.5 HSV-1 mutant, which led to increased CD8^+^ T cell proliferation [136]. In contrast, atg5^−/-^ DCs infected with the γ34.5 HSV-1 mutant did not induce CD8^+^ T cell proliferation [136]. These results indicate that the γ_1_34.5 protein has a role in the inhibition of autophagosome maturation and that this alters the ability of DCs to present antigens to T cells [136]. In addition, an *in vivo* study in mice with a cDC having a conditional deletion in the gene for Atg5 showed impaired CD4^+^ T cell priming after infections with HSV-1 or HSV-2, with the most pronounced defect from Atg5 deletion being the processing and presentation of phagocytosed antigens in MHC-II [137]. Importantly, the Atg5 deletion did not affect the cross-presentation of peptides in the MHC-I molecules [137]. Regarding the role of autophagy in regulating MHC-II-dependent antigen presentation by DCs to CD4^+^ T cells during HSV infection, a study using the herpetic stromal keratitis (HSK) model found that mice with DCs deficient in autophagy (atg5^fl/fl^ CD11c-cre) displayed abrogated CD4^+^ T cell activation, which in turn reduced corneal inflammation in HSK lesions and decreased the severity of the disease, without affecting HSV-1 replication [138].

Taken together, the findings reported above indicate that HSVs infection of DCs overall elicits the inhibition of autophagosome maturation in these cells and impacts the presentation of viral antigens to T cells and their activation.

## Unfolded protein responses in DCs during HSV infections

The cellular responses mounted to deal with unfolded proteins, or protein aggregates within cellular compartments is termed the UPR. This process aims to restore cell homeostasis to avoid endoplasmic reticulum (ER) stress and potential detrimental outcomes for the cells [139]. ER stress occurs, among others, due to a decreased capacity of the cell to carry out protein folding such as after the disruption of Ca^2+^ homeostasis in the ER, increased demands for lipid synthesis in the ER, high protein glycosylation requirements, and increased protein folding needs with few available resources [140–143]. At present, three kinds of ER stress sensors have been described to restore cell homeostasis in mammals (for a detailed review on UPR, we recommend reading Frakes and Dillin, 2017) [144]. Two ER stress sensors are transmembrane proteins located in the ER; one is known as inositol-requiring protein-1α (IRE-1α), which catalyzes the non-canonical splicing of X-box binding protein 1 (XBP-1) mRNA into a constitutively active form XBP-1, and the other response is that mediated by PERK (PKR-like ER kinase) [145–147]. Importantly, XBP-1 regulates several gene targets required for ER homeostasis, such as protein folding genes, ER-associated protein degradation (ERAD), ER protein translocation, and lipid synthesis [146]. The third sensor is termed activating transcription factor 6 (ATF6) located in the Golgi apparatus [148].

In DCs, the UPR play an essential role in the survival and differentiation process of these cells, both under homeostasis and pathological conditions [149]. In homeostasis conditions, the IRE-1α-XBP-1 axis is constitutively activated in iDCs, a process that promotes DC development and survival [150]. Indeed, XBP-1-deficient lymphoid chimeras (XBP-1/RAG-2^−/-^ chimeric mice) have a reduced number of cDCs and pDCs, and low levels of IFN-α are detected [150]. Additionally, gene silencing of XBP-1 in splenic DCs reduces the expression of CD80, CD86, MHC-II and IL-12, and TNF-α cytokine secretion after high mobility group box-1 protein (HMGB1) treatment, a protein that induces the maturation and activation of DCs [151,152]. Consistently, another study also showed that DCs have constitutively activated IRE-1α and XBP-1 in the absence of ER stress and XBP-1 deletion in CD11c^+^ cells produced several phenotypic defects, such as ER homeostasis and CD8a^+^ cDC-mediated antigen presentation [153]. Also, the capacity of DCs to stimulate the proliferation and differentiation of T cells was reduced in the XBP-1-silenced DCs treated with HMGB1 [151]. These effects were explained by a fall in the expression of the receptor for advanced glycation end products (RAGE) on the surface of XBP-1-silenced DCs and induced by HMGB1 stimulation [151].

However, pathological conditions can also induce ER stress, and subsequently initiate UPR responses in DCs, such as in the tumor microenvironment and during virus infections, among others [149]. In tumor cell lines, such as those derived from melanoma, carcinoma, and prostate cancer, the UPR response can function in a cell-extrinsic manner via the molecular transducer transmission of ER stress (TERS) in BMDCs [154,155]. TERS in BMDCs produces the upregulation of the expression of IL-6, TNF-α, IL-23, MCP-1, MIP-1α, and MIP-1β [154]. Also, TERS generates the downregulation of antigen-specific cross-presentation and reduces the effectiveness of cross-priming of CD8^+^ T cells, generating T cell activation without proliferation and suppression of cross-priming by bystander BMDCs [154].

Furthermore, a study reported that HSV-2 infection of BMDCs produces the modulation of the UPR response by altering the splicing of XBP-1 mRNA of the IRE-1α pathway, while ER stress markers and the PERK and ATF-6 signaling pathways were not altered (Figure 3(c)) [43]. Given that the outcome of HSV-2-infected DCs is apoptosis, it is possible that the UPR pathway activated by HSV in these cells may be directly related to cell death, as the activation of IRE-1α may eventually lead to loss of cell viability in certain circumstances [156,157].

In epithelial cells, infection with HSV-1 generates an UPR response that leads to high demands for protein synthesis during viral replication in order to express proteins that make up the capsid, the tegument, and virus envelope glycoproteins [158]. Here, the ICP0 protein of HSV-1 has been reported to act as a potential sensor to modulate the cellular stress response in epithelial cells (HeLa), because ICP0 led to the activation of UPR enhancers during the replication of HSV-1 [158]. In epithelial cells infected with HSV-1, the RNAse activity and the kinase activity of IRE-1α were shown to have different effects over viral replication, with the viral UL41 protein negatively modulating the splicing of XBP-1 and therefore its expression [159]. Finally, the UPR response could play an important role in HSV-mediated inhibition of the capacity of DCs to activate T cells. However, UPR responses have been somewhat poorly studied in the context of HSV infection in DCs.

## HSVs impairs the migration of DCs to lymph nodes

DCs are potent antigen-presenting cells that take up antigens at different sites and then migrate to draining lymph nodes for initiating or regulating T cell responses at these sites. The migration of DCs is a regulated process controlled by several factors, such as chemotactic and non-chemotactic molecules [160]. On the one hand, numerous chemotactic factors, such as C-C chemokine receptors (CCR)1, CCR2, CCR3, CCR5, CCR6, CCR7, CCR9 and CCR10, as well as C-X-C chemokine receptors (CXCR)3, CXCR4 and CXCR5, have been reported to be associated with DC recruitment to periphery tissues and their migration to lymph nodes, where these cells coordinate the activation and regulation of antigen-specific T cell responses [161]. While CCR1, CCR2, CCR5, CCR6, and CXCR1 are downregulated in mDCs, the expression of CCR7 is strongly induced in DCs treated with LPS, CD40L and TNF-α stimuli to promote the migration of uninfected DCs [37,162]. On the other hand, non-chemokine factors, such as bacterial products, danger-associated molecular patterns (DAMPs), complement proteins, and lipids, also are involved in DC migration to lymph nodes [163–165]. HMGB1 remodels the actin-based cytoskeleton of human myeloid DCs through the upregulation of CCR7 and CXCR4 [166]. On the other hand, the presence of the proinflammatory mediator prostaglandin E2 (PGE2) and its recognition by the prostaglandin E2 receptor 2 (EP2) or 4 (EP4) is necessary to elicit a DCs migratory phenotype commanded by CCL21, CXCL12 and C5a secretion [165].

Several studies have shown that HSVs affect DC migration upon their infection. Notably, a study showed that HSV-1-infected MoDCs reduce their migratory capacity toward CCL19 and CXCL12 chemokine gradients by reducing CCR7 and CXCR4 expression [167]. On the other hand, DC migration experiments using CCL19-directed transwell migration showed that HSV-1 and HSV-2 inhibit the translocation of DCs from lower wells to upper wells at 4 hpi [37]. A similar effect was observed with an HSV-1 mutant that had the vhs gene deleted from the genome, in which MoDCs lost their ability to migrate toward a CCL19 stimulus, suggesting that this protein is not essential for the DC migratory process [168]. Importantly, this effect observed with CCL19-direct transwell inhibition migration upon HSV-1 and HSV-2 infections is correlated with a decrease in CCR7 surface expression, as well as CXCR4 in HSV-infected human mDCs [37]. In contrast, cutaneous HSV-2 infection has been reported to upregulate CCR7 expression in CD207^+^ skin murine DCs (LCs and CD207^+^ dermal DCs) [37]. Besides, HSV-2 increases E-cadherin expression in murine LCs after cutaneous infection, a process that also blocks the migration of these cells to lymph nodes [117]. This latter occurs because, in the non-inflamed skin, E-cadherin elicits the interaction of LCs with keratinocytes that generate non-motile cells [117]. Once LCs are activated, they downregulate E-cadherin and its interaction with epithelial cells allowing LC migration to lymph nodes [169]. Another molecule that acts as a factor for CCR7-mediated migration of DC is prostaglandin E2, which acts through the EP2 and EP4 [165]. Interestingly, a study identified that HSV-1-infected human mature MoDCs display a deregulation of EP2 and EP4 mRNA profiles after infection with HSV-1 [168]. However, it is unclear how the deregulation of EP2 and EP4 impact human MoDC migration in the context of HSV infection [168]. On the other hand, CXCR3 chemokine receptors may also be essential for allowing the migration of DCs infected with HSV-2 to lymph nodes, as CXCR3^−/-^ mice showed a significant decrease in the number of both cDCs (B2020-CD11c^+^) and pDCs (B220^+^CD11c^+^) being mobilized to draining lymph nodes in a genital HSV-2 infection model 3 days post-infection [170].

Regarding other adhesion and non-chemotactic factors, it has been reported that infection of mature human MoDCs with HSV-1 or HSV-2 produces a proteasome-dependent degradation of cytohesin-1 interacting protein (CYTIP), a key regulator of DC motility [37,171]. While CYTIP is rapidly degraded in the context of infection, β2 integrins, such as predominantly lymphocyte function-associated antigen 1 (LFA-1), are activated [171]. This study also showed that the impairment in HSV-1-infected DC migration is at least partially mediated by increased adhesion to fibronectin in later time points of infection (16–24 hpi), as CYTIP expression allows the loss of fibronectin adherence in mDCs [171]. Moreover, an increase in integrin-mediated adhesion correlated with the inhibition of the migration of HSV-1-infected human mDCs [172]. Likewise, HSV-2 also inhibited the migration capacity of mDCs by a fast induction of mDCs adhesion by the β2 integrin LFA-1, as determined in an *in vitro* study, which was dependent on chemokine expression [37].

Another host factor that is modulated by HSV infection in DCs is caveolin-1 (Cav-1), an essential structural protein of caveolae that modulates signaling pathways associated with actin cytoskeleton remodeling [173–176]. DCs with a Cav-1 deficiency presented a reduction in their migration capacity toward draining lymph nodes, but no impact was found over their differentiation, maturation, or the capacity to activate CD8^+^ T cells when exposed to LPS and TNF-α [176]. However, Cav-1 deficiency in DCs infected with HSV-1 produced a decrease in the viral loads obtained in the culture supernatants but did not influence DC migration to the lungs [173]. Furthermore, DCs with Cav-1 deleted and infected with HSV-1 showed an increase in the production of inducible nitric oxide synthase (iNOS) and nitric oxide (NO), while WT DCs infected with HSV-1 showed a reduction in the synthesis of these molecules [173]. Interestingly, the decrease in these molecules could be due to the colocalization of Cav-1 with iNOS and HSV-1 in the caveolae of HSV-1-infected DCs [173]. However, the impact of the modulation of iNOS and NO on the function of DCs infected with HSV-1 remains unclear.

Finally, gB and gC interactions with the DC-specific intercellular adhesion molecule-grabbing nonintegrin (DC-SIGN) have been reported to promote HSV-1 infection of iDCs, but it is unknown if this impacts DC migration to lymph nodes [177]. On the other hand, an *in vivo* study showed that HSV-2 inhibits the migration of CD103^+^ dermal DCs (dDCs) from the footpads of mice to draining popliteal LN [117]. Interestingly, the inhibition of DC migration to lymph nodes was shown to be reversed when the animals were inoculated with an HSV-2 gD mutant (ΔgD-2), but not with a gH mutant (ΔgH-2) [43].

## T cell activation by DCs during HSV infection

As discussed above, HSVs have evolved different mechanisms to modulate DC function, leading to cell death and impairments in their migration from the initial site of infection toward the draining lymph nodes. This latter process certainly promotes the priming of virus-specific naïve T cells, which overall could help control infection by further supporting antiviral antibody production by B cells, or the killing of virus-infected cells at peripheral tissues [26]. Importantly, a study has shown that the γ_1_34.5 viral protein of HSV-1 plays an essential role in the inhibition of naïve T cell activation by interfering with the formation of autophagosomes and antigen presentation in DCs, as discussed above [69,131,136]. However, MHC-I cross-presentation by bystander non-infected DCs has been suggested as a way to counteract viral immune evasion and effectively activate CD8^+^ T cell responses against HSVs [178]. CD8^+^ T cell activation by DCs requires the presentation of peptides in the context of MHC-I molecules, with such peptides being processed within the cytosol and ER [179]. Cross-presentation, in contrast, enables the translocation of exogenous peptides incorporated into endosomes onto the MHC-I presentation machinery and involves the ingestion of viral-infected cells, viral debris, or viral proteins by bystander DCs [180]. However, the HSV-1 infected cell protein 47 (ICP47) has been extensively reported to inhibit MHC-I antigen presentation by interacting with the transporter associated with antigen presentation (TAP) and consequently attenuate virus-specific cytotoxic T lymphocytes (CTLs) responses [181].

Noteworthy, there are studies that suggest that some peripheral DCs are stimulated by the acquisition of antigens from the phagocytosis of infected cells undergoing apoptosis, as during skin HSV infections, which then migrate to lymph nodes to activate naïve T cells [182]. Further, such uninfected DCs, when pulsed with HSV-infected apoptotic DCs stimulate HSV-specific CD8^+^ T cells [118]. Once in the lymph node, it has been observed that LN-resident CD8α^+^ DCs are responsible for cross-activating CD8^+^ T cells instead of migrating-DCs [183]. Interestingly, different subtypes of DCs have been isolated from brachial LNs of infected mice at 2 days post-skin infection and co-cultured *in vitro* with HSV-specific gBT-I cells labeled with CFSE to evaluate their proliferation [183]. In such study, it was found that contrary to the classical view that LCs initiate T cell priming at the draining lymph nodes, it was the CD8α^+^ DC subset that activated virus-specific cytotoxic T cells in the brachial lymph nodes 2 days after a flank infection [183]. Importantly, the ability of CD8α^+^ DCs to activate CD8^+^ T cells depended on the migration of peripheral bystander DCs to the lymph nodes, as blocking the migration of DCs with the synthetic prostaglandin analog BW245C, severely impaired antiviral CTL responses [184]. This study argues that the handover of HSV-derived antigens from migrating DCs to resident cells in the lymph nodes is an essential mechanism for priming CD8^+^ T cells by cross-presentation [184]. Furthermore, it has been hypothesized that CD8α^+^ DCs may obtain HSV-derived antigens from migrating DCs via exosomes or through gap junctions [185]. Moreover, a study using gH- and gB-deleted HSV-1 mutants corroborated the ability of CD8α^+^ DCs to cross-present rather than directly present HSV antigens to T cells [186]. *In vitro* experiments evaluated the proliferation of CTLs by CD8α^+^ DCs cells after the exposure of these DCs with cells infected either with gH- or gB-HSV-1 mutants [186]. Because these mutant viruses do not produce infectious progeny, CD8α^+^ DCs were not infected, and the proliferation of CTLs that was observed was produced uniquely by the cross-presentation of antigens and cell fragments from HSV-infected DCs engulfed by CD8α^+^ DCs [186]. Moreover, the ability of CD8α^+^ DCs to effectively activate CTL responses *in vivo* depended on TLR3 expression and TRIF [187]. Mice lacking TRIF or TLR3 showed a lower CTL response and impaired viral clearance at the site of infection after HSV-1 flank infection, as compared with WT or MyD88^−/-^ mice [187].

In addition, a recent study reported that CTL activation does not require infected DCs, as determined by using an approach in which all HSV-infected DCs were depleted [188]. R-DTA mice inoculated with a Cre-recombinase-expressing HSV-1strain KOS virus-induced diphtheria toxin expression and death of infected cells [188]. In this context, uninfected-DC subtypes isolated from the brachial lymph nodes of the infected R-DTA mice and co-cultured with virus-specific CD8^+^ T cells showed that CD8α^+^ DCs were the only DC subtype that induced effective CD8^+^ T cell proliferation, reinforcing the well-known ability of CD8α^+^ DC to cross-present antigens [188]. Moreover, this study revealed that LCs and pDCs were dispensable for T cell priming. Additionally, Irf8^−/-^ mice were severely compromised in mounting an HSV-specific CD8^+^ T cell response after HSV-1 skin infection [188]. As expected, these animals displayed higher viral titers in the skin than WT mice. This result suggests that CD8α^+^ DCs and CD103^+^ DCs, which depend on IRF8 signaling, are required to induce an effective virus-specific CD8^+^ T cell immunity during peripheral HSV-1 infection [188]. Moreover, in an HSV-1 skin infection model, it was shown that localized infection elicits a Th1 response that is primed in skin-draining lymph nodes and involved antigen presentation by migratory dermal and lymph node-resident DCs [189]. This process deteriorated in response to UV-inactivated HSV-1 inoculation, as well as infected mice that lacked CD8α^+^ and CD103^+^ cDC1s [189]. However, pDCs alone failed to induce CTL activation because mice with depleted pDCs displayed increased viral loads and reduced production of IFN-γ by CD8^+^ T cells [190].

Regarding MHC-II-restricted presentation of HSV antigens by DCs, naïve HSV-specific CD4^+^ T cells are reportedly stimulated by dermal CD103^+^ DCs isolated from the lymph nodes draining the primary site of HSV infection, which stimulate their differentiation into effector phenotypes [191,192]. Also, migratory CD103^+^ DCs from LNs elicited a higher expression of Th17 cytokines by CD4^+^ T cells, as compared to CD8^+^ T cells, which was correlated with IL-1β and IL-6 production by CD103^+^ DCs [193]. Although early antigen presentation is carried out by lymphoid-resident DCs that initiate the activation of antigen-specific T cells in the draining lymph nodes, this is insufficient for their clonal expansion. Therefore, migratory dermal DCs must interact with CD4^+^ T cells present in the LNs to induce their appropriate proliferation and differentiation [194]. These HSV-specific CD4^+^ T cell responses have been reported to be important for CTL priming, as well as for the resolution of HSV infection in the skin [195,196]. Moreover, in a cutaneous HSV-1 infection model, the early priming of CD4^+^ T cells involved their clustering with migratory skin DCs. However, CD8^+^ T cells did not interact with migratory DCs [197]. Subsequently, CD8^+^ T cell activation required a later clustering and interaction with LN-resident XCR1^+^ DCs, in which CD4^+^ T cells interacted with these late CD8^+^ T cell clusters together with resident XCR1^+^ DCs [197].

On the other hand, Zhao et al., have reported that after HSV-2 intravaginal infection, viral antigens are presented to CD4^+^ T cells by CD11b^+^ submucosal DCs, instead of LCs or CD8α^+^ DCs, a process that produces the stimulation of IFN-γ secretion [198]. A subsequent study showed that, after viral recrudescence at 5 days post-HSV-1 flank skin infection, another DC subtype participates in the cross-presentation to CTLs, namely CD103^+^ DCs, which are skin-derived DCs that express the langerin-like LC marker. Contrary, all DC subtypes were able to present antigens to virus-specific CD4^+^ T cells at day five after infection in the axillary and brachial lymph nodes [199]. Additionally, several studies have demonstrated the essential role of CD4^+^ T cells in enhancing virus-specific CTL responses following subcutaneous infection with HSV, which is impaired in the absence of CD4^+^ T cells [200,201]. Mixed bone marrow chimeras revealed that the same DCs that activated HSV-specific CD8^+^ T cells *in vivo* required receiving signals from cognate CD4^+^ T cells, with CD8α^+^ DCs also displaying HSV-derived antigens on MHC-class II molecules *ex vivo* [191]. However, the mechanisms used by CD4^+^ T cells to improve the capacity of CD8α^+^ DCs to drive the differentiation of HSV-specific CTLs toward their effector phenotype need to be analyzed in more detail.

Regarding the role of pDCs *in vivo*, the activation of this DC subtype by HSV-1 was shown not only to induce the production of IFN-α by these cells, but also stimulated naïve CD4^+^ T cells and led to the migration of activated T cells to the infection site thanks to the chemokines CCL4 and CXCL10 produced at the site of infection [44]. Moreover, pDC-depleted mice displayed decreased CD8^+^ T cell responses and increased viral loads during cutaneous HSV-1 infection [190]. Those studies showed that although pDCs alone fail to induce virus-specific cytotoxic T cells, it seems that they are able to deliver some help to lymph node DCs [190].

Further, DCs exposed to increased HSV-1 viral titers *in vivo* have been reported to result in gradually decreased Th1 responses, with a decrease in IFN-γ, IL-4 and IL-10, IL-12(p40), and IL-12(p70) expression [202]. On the other hand, the ICP10 HSV-2 protein has been somewhat associated with the inhibition of Th1 responses [203]. In a study by Gyotoku et al., an ICP10 HSV-2 mutant that has the serine-threonine protein kinase (PK) domain of the large subunit of ribonucleotide reductase deleted, elicited an HSV-2-specific Th1 response, which was associated with higher levels of IL-12, HSV-specific IgG2a (Th1), more than IgG1 (Th2) isotypes and higher numbers of CD4^+^ IFN-γ than IL-10 secreting T cells in popliteal lymph nodes [203]. However, the effects of this mutation over the expression of other viral components and, overall, the HSVs replication cycle may have also promoted this type of response.

## Vaccine approaches against HSV: HSV-DC interactions

Due to the key immune-modulatory functions exerted by DCs in the initiation and regulation of antiviral immune responses, these cells have become a focus in vaccination strategies to bestow protective immunity against viral infections [204–206]. Given the capacity of these cells to process pathogen-derived and self-antigens in residing tissues or circulating throughout the body, and present them to T cells, these potent antigen-presenting cells acquire a protagonist role in coordinating optimal antigen-specific immunity [207]. Indeed, to elicit a protective immune response upon vaccination, DC activation has the potential to enact a robust adaptive immune response, either through humoral immunity or cellular immunity, dependent on T CD8^+^ and CD4^+^ T cells [208–211].

Interestingly, the magnitude of the activation of DCs stimulated with virus-based vectors seems to be related to the quality of the immune response elicited and related to the expression of costimulatory molecules in these cells (i.e., CD40, CD83 and CD86), while concomitantly inducing antigen-specific T cell responses [212]. On the other hand, immature or semi-mature DCs have been demonstrated to have a role in inducing tolerogenic environments, which is mediated either through the modulation by exosomes, conventional CD11c^+^ DCs expressing perforin, and contacts with steady-state CD103^+^ DCs, in order to regulate excessive inflammation, autoimmune diseases, or support allograft tolerance [213–216].

At present, there are several existing vaccine formulations against viruses that rely on DC recognition, namely DNA- and RNA-based, combinations of viral glycoproteins with adjuvants, and live-attenuated viruses or viral vectors, among others [217–220]. As expected, each of these vaccine approaches has its advantages and disadvantages compared with each other. For instance, ribonucleic acid vaccines have shown remarkable results *in vitro* [221], as well as in animal models [222] by promoting robust adaptive immunity that could correlate with protection. However, difficulties in implementing them lie in reaching antigen presenting cells, and nanoparticles and lipid vesicles have been tested as effective delivery systems [206,223,224]. On the other hand, the use of single or multimeric antigens in subunit vaccines may require at times additional boosts in order to elicit sufficient stimulation capable of inducing robust immune defenses [225–228]. Alternatively, live-attenuated and vector vaccines are efficient in delivering high quantities and a wide variety of antigens for eliciting immunogenicity. However, depending on the inactivation or attenuation methods, they may put at risk immunosuppressed individuals or eventually revert to virulent forms. Their effectiveness may also be interfered due to preexisting immune responses mounted against whole viruses [229–231].

Regarding HSV-1 and HSV-2, most prophylactic strategies assessed so far, and for decades, have consisted of subunit vaccines (Table 2) [232–234]. Unfortunately, a highly expected Phase 3 clinical study consisting of a subunit vaccine that combined gB and gD viral protein subunits with Alum as an adjuvant showed insufficient protection against infection with HSV-1 and HSV-2 [235]. Therefore, subsequent studies have focused on developing new strategies for boosting immune responses, such as attenuated recombinant HSVs in the hope of eliciting adaptive protective immunity by these means [236]. However, this has proven challenging as HSVs possess numerous molecular determinants that aim to interfere with several functions of innate and adaptive immune cells, including DCs [237]. For instance, some HSV mutants have not elicited the expected protection as vaccines, such as a ΔgH virus [238,239]. Because of the importance of DCs in eliciting and regulating antiviral responses, these cells should be an important focus in vaccination strategies against HSVs. Therefore, understanding the relationship between mutant HSVs and DCs might be at the heart of identifying novel vaccines that could induce robust antiviral adaptive immune responses [43,52]. A favorable interaction between DCs and mutant HSVs for eliciting immunity likely starts with such viruses not killing DCs, which occurs with WT HSV as discussed above, but also has been observed for other HSV mutants, namely HSV-2 viruses lacking either gI, gK or gH [43,52]. Furthermore, it is important to note that UV-inactivated viruses fail to elicit protective immunity [239], but some mutants viruses as HSV-1 γ_1_34.5 mutant virus was shown to be particularly attenuated in DCs and elicit protective immunity *in vivo* in mice [54]. Interestingly, the transfer of DCs from γ_1_34.5 mutant-inoculated mice was capable of providing 90% survival to unvaccinated mice after a lethal challenge [54]. An equivalent result was observed with an HSV-2 mutant virus that lacks gD, which does not kill DCs [43], which has been shown in *in vivo* experiments to be safe, highly immunogenic and elicit protective against the skin, genital and ocular challenges with lethal doses of clinical isolates of HSV-1 and HSV-2 [236,240,241]. It is likely that, among other mechanisms of action, protection by this ΔgD-2 vaccine candidate is given by the ability of the ΔgD-2 virus to enable the survival of DC while promoting viral gene expression and presentation and DC migration to lymph nodes for activation of naïve CD4^+^ and CD8^+^ T cells, in contrast to the disruptive nature of the parental WT virus over DC function [43,242].

**Table 2.** Attenuated herpes simplex virus vaccine candidates that show favorable interactions with DCs

HSVs vaccine (HSVs mutant)	Type of modification (deletion-mutation)	Response (outcome)	Reference
HSV-1 γ34.5	Mutation in the γ34.5 gene.	Complete survival of BALB/c mice from lethal challenge. Associated with DCs conferring protective immunity and the CD4^+^ and CD8^+^ T cell activation.	[54].
HSV-2 ΔgD^−/+gD1^	HSV-2 virus with a deletion of the Us6 gene, but phenotypically complemented with gD on the virion surface.	Confers protection from skin, genital, and ocular challenges with lethal doses of HSV-1 and HSV-2. Associated with increased DC survival, viral gene expression, the promotion of DC migration to lymph nodes and the CD4^+^ and CD8^+^ T cell activation.	[43,236,240,241].

Together, these findings with some particular mutant viruses may indicate that DC survival with viral gene expression may be at the heart of eliciting DCs with T cell activating capacities that are protective against HSVs.

## Concluding remarks

HSV infection of DCs modulates numerous key cellular processes and functions, such as the inhibition of autophagosome maturation, the activation of cell apoptosis, and likely the activation of a detrimental UPR IRE-1α/XBP-1 axis, all together promoting DC death. On the other hand, the infection of DCs by HSV produces the inhibition of the maturation of DCs through the downregulation of costimulatory molecules and reduces their capacity to process and present antigens, which impacts the activation of T cells. In addition, HSV infection causes a significant reduction in the ability of DCs to migrate to the draining lymph nodes, which is necessary for the optimal activation of virus-specific T cells. Because DCs are key cells of the immune system that participate both, in the activation of the immune response against pathogens and in its regulation, all these effects elicited by HSV over DCs will certainly affect the resulting immune response to these viruses, most likely causing suboptimal antiviral responses that could favor the persistence of the viruses and recurrences. Due to the vital role that DCs play in the establishment and regulation of antiviral immune responses, these cells should be central in further vaccine studies, in such a way as to reverse the adverse effects of HSV over these cells for the future development of optimal prophylactic alternatives against HSVs.

## Data Availability

Data sharing does not apply to this article as no new data were created or analyzed in this review.
